# Nurse, midwife and patient perspectives and experiences of diabetes management in an acute inpatient setting: a mixed-methods study

**DOI:** 10.1186/s12912-022-01022-w

**Published:** 2022-09-06

**Authors:** Sara Holton, Bodil Rasmussen, Joy Turner, Cheryl Steele, Deepa Ariarajah, Shane Hamblin, Shane Crowe, Sandy Schutte, Karen Wynter, Ilyana Mohamed Hussain

**Affiliations:** 1 School of Nursing and Midwifery, Deakin University, 1 Gheringhap Street, Geelong, Vic. 3220 Australia; 2 Centre for Quality and Patient Safety Research – Western Health Partnership, Deakin University, 1 Gheringhap Street, Geelong, Vic. 3220 Australia; 3 Department of Public Health, Faculty of Health and Medical Sciences, University of Copenhagen, Blegdamsvej 3B, 2200 Copenhagen, Denmark; 4 Faculty of Health Sciences, University of Southern Denmark, Campusvej 55, 5230 Odense M, Denmark; 5 Nursing and Midwifery, Western Health, PO Box 294, St Albans, Vic. 3021, Australia; 6 Diabetes Education Services, Western Health, PO Box 294, St Albans, Vic. 3021, Australia; 7 Endocrinology and Diabetes, Western Health, PO Box 294, St Albans, Vic. 3021, Australia

**Keywords:** Diabetes Mellitus, Australia, Nurses, Midwifery, Patients, Health Services, Hospitals

## Abstract

**Background:**

In an acute hospital setting, diabetes can require intensive management with medication modification, monitoring and education. Yet little is known about the experiences and perspectives of nursing/midwifery staff and patients. The aim of this study was to investigate diabetes management and care for patients with diabetes in an acute care setting from the perspectives of nursing/midwifery staff and patients.

**Methods:**

A convergent mixed-methods study design. Patients with diabetes (Type 1, Type 2 or gestational diabetes) recruited from a public health service in Melbourne, Australia completed a survey and nurses and midwives employed at the health service participated in focus groups. Descriptive statistics were used to summarise the survey data. Thematic analysis was used for the free-text survey comments and focus group data.

**Results:**

Surveys were completed by 151 patients. Although more than half of the patients were satisfied with the diabetes care they had received (n = 96, 67.6%), about a third felt the hospital nursing/midwifery staff had ignored their own knowledge of their diabetes care and management (n = 43, 30.8%). Few reported having discussed their diabetes management with the nursing/midwifery staff whilst in hospital (n = 47, 32.6%) or thought the nurses and midwives had a good understanding of different types of insulin (n = 43, 30.1%) and their administration (n = 47, 33.3%). Patients also reported food related barriers to their diabetes management including difficulties accessing appropriate snacks and drinks (n = 46, 30.5%), restricted food choices and timing of meals (n = 41, 27.2%). Fourteen nurses and midwives participated in two focus groups. Two main themes were identified across both groups: 1. challenges caring for patients with diabetes; and 2. lack of confidence and knowledge about diabetes management.

**Conclusions:**

Patients and nursing/midwifery staff reported challenges managing patients’ diabetes in the hospital setting, ensuring patients’ optimal self-management, and provision of suitable food and timing of meals. It is essential to involve patients in their diabetes care and provide regular and up-to-date training and resources for nursing/midwifery staff to ensure safe and high-quality inpatient diabetes care and improve patient and staff satisfaction.

**Supplementary Information:**

The online version contains supplementary material available at 10.1186/s12912-022-01022-w.

## Background

The prevalence of diabetes in Australia is increasing and as a result, the number of patients admitted to hospital with diabetes has also risen. It is estimated that approximately 2.5—3 million people in Australia, that is 1 in 10, will be affected by diabetes by 2025 [1]. Approximately 25% of hospital inpatients have diabetes at any point in time [2].

Diabetes care can be complex and may result in prolonged hospital stays, higher patient mortality, and increased cost to health services [3, 4]. Thus, its management in a hospital setting requires intensive management with medication modification and monitoring in order to deliver safe and high-quality care.

In Australia, nurses and midwives are the primary providers of inpatients’ diabetes care. Nursing care of people with diabetes has become increasingly complex and time-consuming [5], and is often undertaken by non-diabetes specialist nursing/midwifery staff [6]. As well as facilitating patients’ food intake, ward nurses/midwives are responsible for conducting and documenting clinical assessments such as monitoring of blood glucose levels which are crucial for treatment decisions made by the medical team. Nurses/midwives are also required to administer, or supervise patient self-administration of, diabetes medication. The increasing diversity of insulin preparations and complexity of insulin regimens places an increased burden on nurses/midwives regardless of their ward specialty due to the need to have a thorough working knowledge of different insulins and their actions [7]. Nurses/midwives may also need to assess each patient’s diabetes knowledge, identify barriers to diabetes self-management such as psycho-social needs, and refer patients to relevant practitioners [7–10]. In acute care settings managing the diabetes care needs of patients can be difficult for nurses/midwives working on busy wards as their roles require clinical decision making about a range of complex medical and surgical conditions in addition to diabetes.

The demands of caring for patients with diabetes can contribute to medication errors and unsafe patient care. An audit conducted in 2018 found that insulin errors were one of the top ten incidents reported in Australian hospitals [11]. Distraction and interruption during medication administration, complexities of calculating appropriate insulin doses, and consideration of patients’ individual needs have all been identified as factors associated with insulin administration errors [12].

Providing care for diabetes patients can therefore be challenging for nurses and midwives who on daily basis may be responsible for one or more diabetes patients each of whom may have diverse clinical needs and management plans.

The aim of this study was to investigate diabetes management and care for patients with diabetes in an acute care setting, including patients’ experiences and perceptions of the diabetes care they received as an inpatient, and nurses/midwives’ experiences and perceptions of caring for patients with diabetes, and the barriers and enablers to providing such care.

## Methods

### Study design

The research used a convergent mixed-methods study design. The study included a patient survey (quantitative data) and focus groups with nurses and midwives (qualitative data).

### Sample, recruitment and procedure

#### Survey (patients)

Patients with diabetes (Type 1, Type 2 or gestational diabetes) aged 18 years or older who at the time of the study were or had been an inpatient at the study health service were invited to participate in the research by members of the research team. Patients were recruited via direct approaches from members of the research team (current patients) and email or SMS to their mobile phone number which included the link to the online survey (previous patients).

Over a third of patients at the study health service have diabetes [2] and almost a quarter of all pregnant women who give birth at the study health service have gestational diabetes and of these women, most (70%) are treated with insulin.

#### Focus group (nurses/midwives)

Nurses and midwives practising at the study health service (*n* = approximately 3,000) were invited by email to participate in a focus group. Nurses and midwives were asked about their experiences and perspectives of caring for patients with Type 1, Type 2 or gestational diabetes. Focus groups were recorded and transcribed verbatim.

As the nurses/midwives invited to participate in the study had specific experiences and knowledge of diabetes care and management, a sample of 10 – 15 nurses/midwives was considered to offer adequate information power [13].

#### Data collection

The study was conducted in late 2020—2021 and data collection was adversely affected by the COVID-19 pandemic. In order to minimise the burden on nurses and midwives and patients during the pandemic, and the impact on essential clinical care, the data collection phase of the project was delayed until after ‘Wave 2’ of the pandemic in Melbourne, Victoria (ie November 2020).

#### Patient survey

Patients were invited to complete a self-report anonymous online survey. The survey was informed by the researchers’ clinical and research expertise, the published literature, and the research questions. It was pretested by a person with diabetes and a diabetes educator; minor changes were made to the question wording and response options as a result. The survey was available in Qualtrics, an online survey tool; and took approximately 15 min to complete. The survey included fixed-response and open-ended questions and assessed respondents’ perceptions, experiences, needs and preferences of diabetes management within an acute care setting. Sociodemographic characteristics (such as age, relationship status, country of birth, language/s spoken at home, highest level of education, and socioeconomic status – health care concession card and residential postcode) and diabetes history (type, age at diagnosis) were also collected (a copy of the survey is included as a supplementary file).

#### Focus group (nurses and midwives)

Separate focus groups were conducted with nurses and midwives who were providing care at the study health service. A discussion guide was developed, informed by the researchers' clinical and research expertise, the published literature relating to diabetes management and nursing/midwifery care in acute care settings, and the research objectives. Nurses’ and midwives' views were sought about their perceptions and experiences of providing care for patients with diabetes, and the barriers and enablers to providing such care (a copy of the focus group discussion guide is included as a supplementary file). The focus groups were held on Zoom due to the COVID-19 pandemic and facilitated by members of the research team, audio-recorded with participants’ permission and transcribed verbatim.

### Data analysis

The qualitative data from the focus groups and the quantitative data from survey were collected concurrently but analysed separately. The results were considered together in order to address the study’s objectives [14].

### Quantitative (survey) data

Descriptive statistics were used to describe and summarise all study variables. Chi-square tests were used to assess differences between survey respondents with type 1 and type 2 diabetes. Quantitative data analysis was conducted using IBM SPSS Statistics.

### Qualitative (focus group) data

The free-text comments from the survey (patients) and the transcripts of the focus groups (nurses and midwives) were analysed separately using thematic analysis techniques commonly practised in qualitative research [15]. As identified by Braun and Clarke [15] this consists of six phases. Phases 1 and 2: Transcripts are repeatedly read and reread and coded. Phases 3–5: Codes are grouped into meaningful categories that describe how participants talked about the topics, including contradictions and exceptions. Themes are created, named and defined in order to explain and interpret the content. Examples of the identified themes are selected in the final phase (phase 6) and related back to the research objective. The analysis was conducted by members of the research team and interpretations were discussed within the research team until consensus is reached.

Survey respondents’ free-text comments and illustrative quotes from the focus group participants are included in the Results and as a supplementary file.

## Results

### Patient survey

#### Respondents’ sociodemographic characteristics

The survey was completed by 151 patients. The average age of the survey respondents was mid-fifties, and more than half were born in Australia and had a post-secondary school qualification. Almost two-thirds had a partner and a healthcare concession card. About a third had private health insurance and a quarter spoke another language than English at home (Table 1).

**Table 1:** Survey (patient) respondents’ sociodemographic characteristics

Characteristic	Study sample *n* = 151
Age mean (range, SD)	54.9 (21–83, 14.0)
Born in Australia	85 (56.3%)
Aboriginal or Torres Strait Islander	4 (2.7%)
Speaks language other than English at home	39 (26.0%)
Post-secondary school qualification	80 (53.7%)
Relationship status—partnered	95 (62.9%)
Has a healthcare concession card	94 (62.3%)
Has private health insurance	48 (31.8%)

#### Respondents’ diabetes history, medication and management

Almost three-quarters of the respondents reported that they had type 2 diabetes and the average age at diagnosis was 40.4 years. Most respondents reported that they were able to self-manage their diabetes at home and just less than half had insulin treated diabetes (Table 2).

**Table 2:** Survey (patient) respondents’ diabetes characteristics and management

Characteristic	Study sample *n* = 151
Type of diabetes mellitus	
*Type 1*	40 (26.5%)
*Type 2*	110 (72.8%)
*Gestational Diabetes Mellitus*	1 (0.7%)
Insulin treated diabetes mellitus	
*Yes*	67 (45.6%)
*No*	80 (54.4%)
Age at diagnosis, Mean (range, SD) (*n* = 133)	40.4 (2–69, 16.7)
Self-manage diabetes at home	
*Yes, all of the time*	127 (86.4%)
*Most of the time*	13 (8.8%)
*No, I have assistance at home*	7 (4.8%)
Diabetes medications	
*Rapid-acting insulins*	31 (20.5%)
*Long-acting insulins*	13 (8.6%)
*Combination insulins*	9 (5.9%)
*Insulin (type not specified)*	11 (7.3%)
Biguanides	60 (39.7%)
Dipeptidyl peptidase-4 (DPP-4) inhibitors	19 (12.6%)
Glucagon-like peptide-1 receptor agonists	10 (6.6%)
Sodium-glucose transporter (SGLT) 2 inhibitors	7 (4.6%)
Sulfonylureas	10 (6.6%)

Across the sample of survey respondents at least 30 different medications to manage diabetes including insulins (rapid-acting, long-acting and combination), biguanides (such as Metformin), dipeptidyl peptidase-4 (DPP-4) inhibitors, glucagon-like peptide-1 receptor agonists, sulfonylureas and sodium-glucose transporter (SGLT) 2 inhibitors were identified (respondents may be taking more than one type of medication and therefore, the percentage counts in Table 2 may not equal 100) (Table 2).

#### Inpatient diabetes management experiences and preferences

**Fig. 1:**
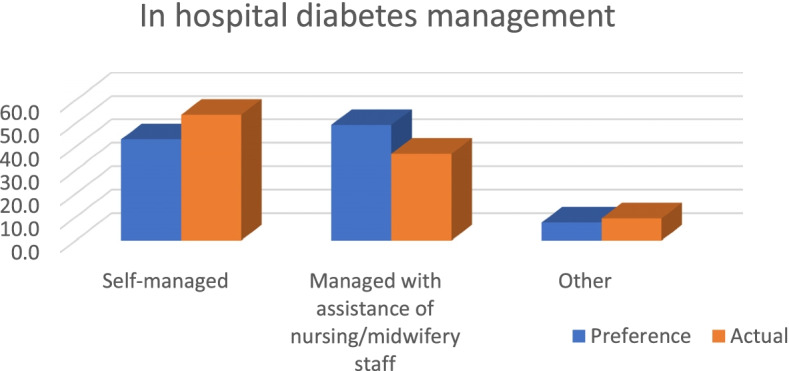
Survey respondents’ in hospital diabetes management (preference vs actual)

More than half of the respondents had been a patient in hospital in the previous 12 months and had self-managed their diabetes during their inpatient stay whilst over a third stated that they had needed assistance to manage their diabetes while in hospital. Despite a third of respondents reporting that they had discussed their diabetes management with the nursing/midwifery staff whilst they were in hospital, almost one in five stated that their discussion with the nurse/midwife was not sufficient or they would have liked to have discussed their diabetes management with a nurse/midwife (Table 3). Almost half of the respondents indicated that their preference was to manage their diabetes with the assistance of the nursing/midwifery staff when they were an inpatient; yet only about a third reported that they had actually received assistance during their previous hospital admission (Table 3 and Fig. 1). There were no significant differences between respondents with Type 1 and Type 2 diabetes in terms of whether they had discussed their diabetes management with nursing/midwifery staff (*p* = 0.055), their preference to manage their diabetes in hospital (*p* = 0.893) and how it was actually managed (*p* = 0.100).

*“If I am able, I would like to be in charge of my own diabetes but in saying that I’d like the nurses and doctors to be looking over to help out if I need help.”* (Survey respondent, Type 1 diabetes)

**Table 3:** Survey (patient) respondents’ inpatient diabetes management experiences and preferences

Diabetes management experience	Study sample *N* = 151
Last inpatient episode	
*Less than 6 months ago*	35 (23.5%)
*6–12 months ago*	52 (34.9%)
*More than 12 months ago*	62 (41.6%)
In hospital diabetes management preference	
*Self-managed*	62 (43.1%)
*Managed with assistance of nursing/midwifery staff*	71 (49.3%)
*Other*	11 (7.6%)
In hospital diabetes management	
*Self-managed*	80 (53.7%)
*Managed with assistance*	55 (36.9%)
*Other*	14 (9.4%)
Discussed diabetes management with nursing/midwifery staff in hospital	
*Yes, completely*	47 (32.6%)
*Yes, to some extent but not enough*	28 (19.4%)
*No, would like to*	25 (17.4%)
*No, don’t want to*	21 (14.6%)
*Can’t remember/Not sure*	23 (16.0%)
Satisfaction with diabetes care in hospital	
*Very satisfied*	49 (34.5%)
*Satisfied*	47 (33.1%)
*Neither satisfied nor dissatisfied*	35 (24.6%)
*Dissatisfied*	8 (5.6%)
*Very dissatisfied*	3 (2.1%)
Knowledge of diabetes care ignored by nursing/midwifery staff in hospital	
*Yes, always*	11 (7.9%)
*Sometimes*	32 (22.9%)
*No, never*	97 (69.3%)
Knowledge of diabetes care assessed by nursing/midwifery staff in hospital	
*Yes, always*	55 (37.9%)
*Sometimes*	46 (31.7%)
*No, never*	44 (30.3%)
Experienced hypo/hyperglycaemia last time in hospital	
*Yes*	37 (25.7%)
*No*	107 (74.3%)
Management of hypo/hyperglycaemia by nursing/midwifery staff	
*Very good*	9 (27.3%)
*Good*	7 (21.2%)
*Acceptable*	9 (27.3%)
*Poor*	6 (18.2%)
*Very poor*	2 (6.1%)
Experienced diabetes medication error in hospital	
*Yes*	12 (10.0%)
*Not sure*	20 (16.7%)
*No*	88 (73.3%)

Over two-thirds of the respondents were very satisfied or satisfied with the diabetes care they had received as an inpatient and reported that their knowledge of their diabetes care and management was assessed by the nursing/midwifery staff in hospital (Table 3). There was no significant difference in the level of satisfaction with diabetes care received in hospital between respondents with Type 1 diabetes and those with Type 2 (*p* = 0.067).

*“I felt my nurses and midwives were really good this time with my diabetes and letting me be in control. So nice to see!”* (Survey respondent, Type 1 diabetes)

About a third felt that the nursing/midwifery staff ignored their own knowledge of their diabetes care and management (Table 3). There was no significant difference between respondents with Type 1 and Type 2 diabetes (*p* = 0.156).

*“Some nurses were diligent in checking my blood sugar levels, others didn't check it at all. None of the doctors wanted to discuss it at all. I had an argument every day with the nurses about what medication I was taking (or not taking).”* (Survey respondent, Type 2 diabetes)

A quarter of the respondents stated that they had experienced hypo/hyperglycaemia the last time they were in hospital. Of these, just under half thought that the management of their hypo/hyperglycaemia by the nursing/midwifery staff was very good or good. One in ten of the respondents reported that they had experienced a medication error whilst an inpatient (Table 3). There was no significant difference in the proportion of respondents with Type 1 diabetes and those with Type 2 who had experienced a medication error (*p* = 0.401).

Only about a third of respondents felt that hospital nurses and midwives have a good understanding of the different types of insulin and how different types of insulin are administered (Table 4). Respondents with Type 2 diabetes were significantly more likely to believe that nurses and midwives had a good understanding of the different types of insulin (30.8% vs 28.2%; *p* < 0.001) and how they are administered (37.1% vs 23.1%; *p* < 0.001) than those with Type 1 diabetes.

*“A lot of the nursing staff didn't have a lot of knowledge about CGM and insulin pumps.”* (Survey respondent, Type 1 diabetes)

**Table 4:** Survey (patient) respondents’ perceptions of nurses’/midwives’ diabetes management knowledge

Nurses’ and midwives’ have a good understanding of …	Study sample *N* = 151
Different types of insulin	
*Yes*	43 (30.1%)
*Not sure*	83 (58.0%)
*No*	17 (11.9%)
How different types of insulin are administered	
*Yes*	47 (33.3%)
*Not sure*	81 (56.3%)
*No*	15 (10.4%)

#### Perceived barriers and enablers to diabetes management

Although half of the respondents felt that the monitoring of their blood sugar levels, administration of insulin and mealtimes were well co-ordinated while they were in hospital, respondents with Type 2 diabetes were significantly more likely to report this than those with Type 1 diabetes (66.3% vs 40.0%, *p* = 0.021). Many respondents reported barriers to their diabetes management. These were mostly about food and included difficulties accessing appropriate snacks and drinks, restricted food choices, the timing of meals, and being unable to determine the amount of carbohydrate in food (Table 5).

*“[I would prefer to] have my medications with me as [I] often had to wait for medications when meals were served that needed to be taken prior to meals not after.”* (Survey respondent, Type 1 diabetes)

*“Missed snacks leading to hypos.”* (Survey respondent, Type 2 diabetes)

*“Food menus not having any carb details on them at all.”* (Survey respondent, Type 1 diabetes)

**Table 5:** Survey (patient) respondents’ perceived barriers and enablers to diabetes management

Barriers and enablers	**Study sample** ***N* = 151**
Monitoring of blood sugar levels, administration of insulin and mealtimes well-coordinated in hospital	
Yes, always	69 (58.0%)
Sometimes	37 (31.1%)
No, never	13 (10.9%)
Last time in hospital experienced …	
Not having easy access to appropriate snacks and drinks	46 (30.5%)
Restricted food/menu choices and timing	41 (27.2%)
Not knowing how diabetes would be managed whilst in hospital	26 (17.2%)
Unable to determine the amount of carbohydrate in food	24 (15.9%)
Concerns that the nursing/midwifery staff did not have sufficient expertise in diabetes	21 (13.9%)
Concerns that nursing/midwifery staff did not understand diabetes needs	16 (10.6%)
Concerns about having insulin taken away on admission to hospital	15 (9.9%)
Feelings of loss of control	14 (9.3%)
Lack of support from nursing/midwifery staff to self-manage diabetes	11 (7.3%)
Feeling unsafe about diabetes care	7 (4.6%)
Different opinions about diabetes management by nursing/midwifery staff last time in hospital	
Yes, often	11 (9.3%)
Yes, sometimes	33 (28.0%)
No	74 (62.7%)

Only a small proportion of respondents were concerned that hospital nursing/midwifery staff did not have sufficient diabetes expertise or understanding of their diabetes needs whilst very few experienced a lack of support from nursing/midwifery staff to self-manage their diabetes or felt unsafe about their diabetes care whilst in hospital (Table 5).

*“I was extremely pleased with my overall care whilst I was in hospital. The nurses were absolutely magnificent. In terms of diabetes knowledge though, I found most nurses didn’t have much of a clue about it. This didn’t impact me too much because I manage my own diabetes care and prefer it this way, and I took it as an opportunity to educate the staff on things like my insulin pump and continuous glucose monitor.”* (Survey respondent, Type 1 diabetes)

#### Focus groups – nurses and midwives

Fourteen nurses and midwives employed at the study health service participated in one of two focus groups. The mean duration of the focus groups was 44.3 min (range: 40.6 – 48.1 min). Two main themes were identified across both groups: 1. challenges caring for patients with diabetes; and 2. lack of confidence and knowledge about diabetes management.

#### Theme 1: challenges caring for patients with diabetes

The nurses and midwives who participated in the focus groups identified several challenges caring for patients with diabetes. Their main concerns were associated with managing increased patient acuity, difficulties keeping updated about new diabetes medications, the additional time required to care for diabetes patients who often have complex care needs, and differences in patients’ abilities to self-manage their diabetes.

The focus group participants indicated that patient acuity has increased, and this often made it difficult to provide suitable care to all their patients with diabetes due to their complex care needs. They also reflected that the amount of support and resources for nurses and midwives has not increased in line with patient acuity.

“Like resources and support and you know, it's a recognition of the acuity of because we never really thought about it being such a problem.” (Nurse)

*“I’ve been almost 20 years at [the study health service] and the patient cohort has grown so much, and the actual complexity of our patients has increased tremendously, and that amount of diabetes educator input we had has not increased exponentially like that as well.”* (Nurse)

Nurses and midwives discussed their high workload and the additional time that is required to appropriately care for patients with diabetes. The participants were aware of the care needs of diabetes patients but reported that they often faced challenges providing the appropriate care including not always having sufficient or easy access to the required resources such as blood glucose monitors (if patients do not have their own) and the additional time required to monitor patients’ blood sugar levels.

*“We need a bit more guidelines as to where we can get equipment like [a glucometer] and things like that [if patients do not have their own].”* (Nurse)

*“[Caring for patients with diabetes] actually takes the nursing staff a lot of time and it's challenging.”* (Nurse)

Participants also reported that they experienced difficulties ensuring patients received suitable meals/snacks that arrived at appropriate times given their medications or insulin or blood sugar levels.

*“ … the meal times when, like lunch comes, and dinner and breakfast will come around at a certain time, but then … you get busy and then you miss the sugar [monitoring].”* (Midwife)

*“[Patients with diabetes often] don’t get like a proper meal, they’ll get pasta, potatoes … so the food’s wrong.”* (Midwife)

The participants reported that although some patients (particularly those who have had diabetes for many years) are able to effectively self-manage their diabetes while in hospital, others require assistance and support from nurses or midwives.

*“It's easier for us if [patients] have their own insulin and their own pump and they know, because they use it, they’ll show us what it is and show us their units, and they give it to themselves. It's much easier than like us doing it all the time, particularly if they’re having to do it every meal and then they're having sugars as well, like that’s a lot of work for us.”* (Midwife)

*“Some patients are really good [at managing their diabetes], some need a lot of prompting.”* (Midwife)

*“I think definitely the [patients] with type 1 diabetes are much [better at self-managing their diabetes] obviously because they’ve had it their whole life and [we] are a lot more confident to just let them do their thing, particularly if they have pumps, … So I think that there are some [patients] that are really like independent like that, especially type 1 … they know what their body is like, it's easier to listen to them. But then there's other [patients] that are like you know newly diagnosed or just have insulin, they need more support from us.”* (Midwife)

The participants also highlighted that they often need to spend additional time with patients whose medical conditions may have changed their ability to self-manage their diabetes.

*“Depends sometimes what happens is these patients have like had type 2 diabetes for 20 years, but then they come in [to hospital] with a stroke, now their cognition is all changed, … and we need to teach them.”* (Nurse)

### Theme 2: Lack of confidence and knowledge about diabetes management

Nurses and midwives stated that they often lacked confidence or knowledge about diabetes management, and would benefit from further education.

*“Insulin itself not so much, I think we’re all quite good at it … but we could I suppose get more insight and education …”* (Midwife)

*“I think there are actually newer [diabetes medications], and I think that we definitely need more education about how they work as well.”* (Nurse)

*“If the person has already eaten, and you think okay should I give the medicine, should I not give the medicine, what do I do here, you know?”* (Nurse)

The participants identified educational needs about diabetes care and management, for example a need for ongoing education, a variety of diabetes resources and a diabetes patient care checklist.

*“People [referring to staff] are asking for more information. We used to have a ‘diabetes resource person’ coming in to provide regular update ….”* (Nurse)

*“I think that we definitely need more education about how they [insulin and other medication] work as well general education about diabetes management.”* (Nurse)

### Summary

Comparison of the survey and focus group findings indicates similarities among patients and nurses and midwives in terms of their preference and ability to self-manage diabetes, diabetes knowledge and understanding, and barriers to optimal diabetes management in hospital (Table 6).

**Table 6:** Summary of main study findings

Overall study themes	Patients	Nurses and midwives
Ability to self-manage diabetes in hospital	● Want to discuss their diabetes management with nurses/midwives ● Want to manage their diabetes with the assistance of nurses/midwives	● Patients differ in ability to self-manage diabetes; impacted by patient acuity
Diabetes knowledge and awareness	● Usually assessed by nurses/midwives when in hospital ● Nurses/midwives sometimes ignore patient’s diabetes expertise and knowledge ● Think nurses/midwives lack understanding of different types of insulins/diabetes medications	● Reported lack of confidence and knowledge gaps about diabetes care and medications ● Identified need for diabetes education and training
Barriers to optimal diabetes management in hospital	● Can be difficult to access suitable food at appropriate times when in hospital	● Often lack of suitable food choices for diabetes patients ● Caring for diabetes patients can be complex ● Additional time required to care for patients with diabetes ● Do not always have access to required resources (e.g. blood glucose monitors)

## Discussion

This study investigated the perspectives and experiences caring for patients with diabetes in acute care settings from the perspectives of both patients and nurses and midwives. The findings indicate that most patients with diabetes were satisfied with the diabetes care they received as a hospital inpatient. Nevertheless, both patients and nurses and midwives reported challenges managing patients’ diabetes and providing safe and high-quality diabetes care including ensuring optimal self-management, provision of suitable food, timing of meals, and a nursing/midwifery workforce with adequate diabetes knowledge and education.

People with diabetes often have the most expertise in managing their diabetes and greater knowledge and experience of administering their medications than hospital nursing/midwifery staff [16–18]. Although most of the patients surveyed in this study reported that they were able to effectively self-manage their diabetes at home, only just over half did so whilst they were in hospital. Patients stated a preference to discuss their diabetes management with nursing/midwifery staff and self-manage while in hospital but would appreciate nursing/midwifery staff assistance if required.

Most patients surveyed in this study were satisfied with the diabetes care they had received in hospital, but a considerable proportion felt that the nursing/midwifery staff had ignored their knowledge and expertise or lacked understanding of their diabetes medications and administration. Providing care for diabetes patients can be challenging for nurses and midwives who may be responsible for one or more diabetes patients. The nurses and midwives who participated in this study identified that caring for patients with diabetes was time consuming and complex. Other studies have also identified that nurses in acute care settings often have knowledge gaps about diabetes care, medications and self-management [7, 19, 20], especially those who work mainly in specialised areas for example, cardiac nursing [21]. The findings of these studies and the current one indicate that further ongoing education of nurses and midwives has the potential to improve their knowledge and care of inpatients in acute care settings who have diabetes [7, 22].

One of the major challenges identified by both nurses/midwives and patients for safe diabetes self-management for hospital inpatients was a lack of suitable food choices and the timing of meals. Patients and nurses/midwives agreed that it was often difficult to co-ordinate blood glucose monitoring, food timing and administration of insulin. Other studies have also reported ill-timed insulin administration and meal delivery in acute care settings [5] and highlighted the negative effect of these on patient satisfaction [23].

### Strengths and limitations

This was a small cross-sectional study which recruited participants from one metropolitan health service in Melbourne, Australia. Therefore, the participants’ perspectives and experiences may not reflect those of diabetes patients and nurses and midwives in other settings. The patient survey was only available online due to COVID-19 infection control and prevention restrictions at the participating health service and accordingly, patients with higher levels of education and English skills may have been more likely to complete it. Focus groups were also held online (Zoom) due to COVID-19 restrictions. A strength of this study was the inclusion of both patients and clinicians. This enabled a wider investigation about diabetes management in an acute care setting. The study design which included use of different methods is a strength of this study and expands the breadth, depth, and range of the research, resulting in more comprehensive results. The survey enabled data to be collected from a large number of patients and the focus groups captured the richness of nurses’ and midwives’ experiences and perceptions. The use of mixed-methods provided a more in-depth understanding of diabetes management in acute care settings from the perspectives of both patients and nurses/midwives.

### Implications for clinical policy and practice

The findings of this study have implications for clinical policy and practice and can be used to inform the development of clinical care guidelines and additional nurse/midwife training and education to enable the delivery of optimum nursing/midwifery care improvement patient safety and experience.

#### Diabetes self-management in hospital

Diabetes is a complex and chronic condition that requires effective self-management by the individual. Appropriate self-management can have a positive impact on the health outcomes of people with diabetes [24] and improve patient safety and satisfaction in hospital [18, 25]. The findings of this study reflect the recommendations of others [18, 25] that patients with diabetes should be supported by nursing/midwifery staff to self-manage their diabetes in hospital where appropriate. Health service policies and practices should provide clear and patient-centred guidelines for diabetes self-management including medication/insulin administration and regular patient assessment considering changing clinical circumstances to ensure the capability and willingness to self-mange whilst in hospital.

#### Education for nursing/midwifery staff

The findings of this study highlighted the need for nursing/midwifery staff to receive regular updated training and education about diabetes care, management and medications. The increasing diversity of diabetes medications including insulin can make it difficult for nurses and midwives to maintain up-to-date knowledge and provide safe and high-quality care. Evidence suggests that education about diabetes for nursing staff in hospitals can improve diabetes management including glycaemic control and hypoglycaemia treatment [6].

#### Suitable food options and timing of meals

Participants in this study (both patients and nurses/midwives) reported that access to suitable food choices and the timing of meals were often barriers to effective diabetes management. Health services should ensure that food options and the timing of meals are appropriate for patients with diabetes and provide details about the carbohydrate content of individual food items [25].

## Conclusion

The findings of this study indicate that overall, most patients with diabetes are satisfied with the diabetes care they received as a hospital inpatient. However, both patients and nursing/midwifery staff reported challenges in providing this care. These challenges included an increase in patient acuity, complex diabetes management including medication regimes, and both patient and nursing/midwifery staff diabetes knowledge deficits. The complexity and lack of patient self-management of diabetes during admission, and the provision of suitable food and timing of meals was also highlighted.

Involving the patient in their diabetes care, including self-management where appropriate, and providing regular and up-to-date training and resources for nursing/midwifery staff are likely to result in safe and high-quality inpatient diabetes care and improve patient and staff satisfaction.

## Supplementary Information

**Additional file 1.**

**Additional file 2.**

**Additional file 3.**

## Data Availability

All data generated or analysed during this study are included in this published article and supplementary files.
