# Supplementary material for: Nurse, midwife and patient perspectives and experiences of diabetes management in an acute inpatient setting: a mixed-methods study
Source: BMC Nurs. 2022 Sep 6;21:249. doi: 10.1186/s12912-022-01022-w (PMC9446645; doi:10.1186/s12912-022-01022-w)
Supplement: Supplementary file 1 — Additional file 1. [file 12912_2022_1022_MOESM1_ESM.pdf]

Thursday, 16 January 2020
2:05 PM

English

Default Question Block

Improving Diabetes Management in the Acute Inpatient Setting

Patient survey

Thank you for your interest in the research study "Improving Diabetes Management in the Acute Inpatient Setting". The study has been approved by the Western Health Low Risk Ethics Committee and the Deakin University Human Research Ethics Committee.

The aim of this study is understand patients' experiences and perceptions of the diabetes care they have received as an inpatient.

The study participant information sheet tells you more about the study and can be accessed via this link: [INSERT LINK TO PICF]

Please read through the participant information sheet.

Please do not write your name on this survey.

There are no right or wrong answers to the questions in this survey. We are simply interested in your experiences, thoughts and opinions. If you are unsure about how to answer a question, please mark the response which corresponds most closely to how you feel.

The survey will take approximately 15 minutes to complete.

Your completion of the survey indicates your consent to participate in the study.

Thank you for your participation. If you have any questions about the study please contact Dr Sara Holton at email: WHDeakinPartnership@wh.org.au.

Section 1. Some questions about you

What was your age at your last birthday?

____

What is the postcode where you live?

____

Are you Aboriginal or a Torres Strait Islander?

- ○ Yes
- ○ No

In which country were you born?

[ ]

Do you speak a language other than English at home?

- ○ No
- ○ Yes (please specify) [ ]

What is the highest level of education you have completed so far?

- ○ Primary school
- ○ Partially completed secondary school (less than Year 12)
- ○ Completed secondary school (Year 12)
- ○ Trade/apprenticeship (eg hairdresser, chef)
- ○ Certificate/diploma (eg child care, technician)
- ○ Bachelor degree
- ○ Higher university degree (eg Master's, PhD)

Which of these best describes your current relationship status?

- ○ Married (in a registered marriage)
- ○ Living with a partner in a relationship
- ○ I have a partner I don't live with
- ○ Separated or divorced
- ○ Widowed
- ○ Single
- ○ Other (please specify) [ ]

Do you have a healthcare concession card?

- ○ Yes

○ No

Do you have private health insurance?

○ Yes
○ No

What type of diabetes mellitus do you have?

○ Type 1
○ Type 2

Do you have insulin-treated diabetes?

○ Yes
○ No
○ I don't know

How old were you when you were diagnosed with diabetes?

What medications do you currently use for your diabetes? Please also provide the dosage and how often you take the medications.

Section 2. Your inpatient diabetes management experiences and preferences

When was the last time you were an inpatient at a Western Health hospital (eg Footscray Hospital, Sunshine Hosptial)?

○

- less than 6 months ago
- 6 - 12 months ago
- More than 12 months ago

How did you manage your diabetes the last time you were in hospital?

- Self-managed (I looked after my diabetes myself)
- Managed with the assistance of nursing/midwifery staff
- Other (please specify)

Did you discuss the best way to manage your diabetes while you were in hospital with the nursing/midwifery staff the last time you were in hospital?

- Yes, completely
- Yes, to some extent but not enough
- No, but I would have liked to
- No, but I did not want to
- I can't remember / Not sure

How would you like your diabetes to be managed while you are in hospital?

- Self-managed
- Managed with assistance of nursing/midwifery staff
- Other (please specify)

What would make it easier for you to manage your diabetes while you are in hospital?

Do you think nurses/midwives at Western Health have a good understanding of the different types of insulin?

- Yes
- Not sure
- No

Do you think nurses/midwives at Western Health have a good understanding of how the different types of insulin are administered?

- Yes
- Not sure
- No

Do you self-manage your diabetes at home?

- Yes, all of the time
- Most of the time
- No, I have assistance at home

How satisfied are you with the diabetes care you received the last time you were in hospital?

- Very satisfied
- Satisfied
- Neither satisfied or dissatisifed
- Dissatisifed
- Very dissatisfied

Do you think your knowledge of your diabetes care was ignored by the nurses/midwives the last time you were in hospital?

- Yes, always
- Sometimes
- No, never

Was your knowledge of your diabetes care assessed by the nurses/midwives the last time you were in hospital?

- Yes, always
- Sometimes
- No, never

Did you experience hypo- or hyperglycemia the last time you were in hospital?

- Yes
- No

How was your hypo- or hyperglycemia handled by the nursing/midwifery staff?

- Very Good
- Good
- Acceptable
- Poor
- Very poor

Did you experience any medication errors in relation to your diabetes management the last time you were in hospital?

- Yes
- Not sure
- No

Section 3. Barriers and enablers to diabetes management

Do you think that the monitoring of your blood glucose levels, administration of your insulin and and mealtimes were well-coordinated the last time you were in hospital?

- ○ Yes, always
- ○ Sometimes
- ○ No, never

Do you experience any of the following the last time you were in hospital? (select all that apply)

- ☐ feelings of loss of control
- ☐ concerns that the nursing/midwifery staff did not have sufficient expertise in diabetes
- ☐ not knowing how your diabetes would be managed whilst you were in hospital
- ☐ restricted food/menu choices and timing
- ☐ lack of support from nursing/midwifery staff to self-manage your diabetes
- ☐ feeling unsafe about your diabetes care
- ☐ concerns that the nursing/midwifery staff did not understand your needs (in relation to managing your diabetes)
- ☐ unable to determine the amount of carbohydrate in your food
- ☐ not having easy access to appropriate snacks (food) and drinks
- ☐ concerns about having your insulin taken away from you on admission to hospital

Sometimes, one nurse/midwife will say one thing and another will say something quite different about the best way to manage your diabetes whilst you are in hospital. Did this happen to you the last time you were in hospital?

- ○ Yes, often
- ○ Yes, sometimes
- ○ No

Section 4. Anything else?

Have we missed anything? If you have anything else you would like to tell us about diabetes care/management in hospital, please write in the box below.

Would you be interested in being involved in the second part of this study?

Once we have analysed the results of this study, we will use the findings to develop education and training resources for nurses and midwives. If you would like to be involved in designing and testing these resources please email your name and address to: WHDeakinPartnership@wh.org.au

Please note that your name and email address cannot be linked to your responses to the survey questions, they are anonymous.

Thank you for completing this questionnaire.

Your responses will contribute to understanding the diabetes management needs and preferences of patients receiving care at Western Health and improved patient care, experience and outcomes; and efficient nursing/midwifery practice.

Deakin University CRICOS Provider Code 00113B.

Powered by Qualtrics
