# Supplementary material for: Nurse, midwife and patient perspectives and experiences of diabetes management in an acute inpatient setting: a mixed-methods study
Source: BMC Nurs. 2022 Sep 6;21:249. doi: 10.1186/s12912-022-01022-w (PMC9446645; doi:10.1186/s12912-022-01022-w)
Supplement: Supplementary file 2 — Additional file 2. [file 12912_2022_1022_MOESM2_ESM.docx]

**Improving diabetes management in the acute inpatient setting**

**Focus group – Nurses/Midwives**

**Discussion Guide**

*[Thank participants for volunteering; introduce self.]*

*[Go through informed consent process.]*

*[Discuss demographic survey; process for obtaining summary of results]*

*[Reminder that can withdraw at any time, and can choose not to contribute to particular topics.]*

*[Talk about discussion process:*

*Confidentiality;*

*opportunity for all to ‘speak’;*

*no right or wrong answers]*

“As you know, the purpose of our study is to learn understand and address the burden of providing nursing care for insulin-requiring diabetes patients in acute settings.”

- First, are there any comments you’d like to make or stories you’d like to tell about your experiences and perceptions of caring for patients who have diabetes?
- How much of your time is taken administering insulin and doing other diabetes/insulin associated tests?
- Do you assess patient’s knowledge of their diabetes care/management?
- Do you think nurses/midwives at WH have a good understanding of the different diabetes treatment regimens and insulins/medications?
- Do you think that nurses/midwives have adequate knowledge and skills to provide safe/quality care for patients with diabetes?
- Do you think nurses/midwives receive adequate training about caring for patients with diabetes?
  - What is done well?
  - What could be improved?
- Would you be comfortable letting a patient self-manage their diabetes whilst in hospital?
- What do you think are the barriers and enablers to providing care in acute care settings for diabetes patients who require insulin? Eg
  - Staff knowledge
  - Staff confidence
  - Staff workload
  - Staff turnover
  - Use of bank/agency staff
  - Patient knowledge
  - Lack of diabetes educators out of hours
  - Opportunities for staff development/training about diabetes
- What do you think could be done to improve diabetes management for patients while they are in hospital? Eg
  - glycaemic control, dosing and coordination of mealtimes etc
- Is there anything else that you would like to mention that we haven’t talked about?
