# Supplementary material for: Nurse, midwife and patient perspectives and experiences of diabetes management in an acute inpatient setting: a mixed-methods study
Source: BMC Nurs. 2022 Sep 6;21:249. doi: 10.1186/s12912-022-01022-w (PMC9446645; doi:10.1186/s12912-022-01022-w)
Supplement: Supplementary file 3 — Additional file 3. [file 12912_2022_1022_MOESM3_ESM.docx]

Supplementary Table 1: Survey (patient) respondents’ free-text comments

| **Main theme** | **Sub-theme** | **Survey respondent free-text comments** |
| --- | --- | --- |
| **Managing diabetes in hospital** | | |
|  | ***Medication management preferences*** | |
|  |  | - Having my medications with me as often had to wait for medications when meals were served that needed to be taken prior to meals not after. |
|  |  | - If the medication wasn’t kept in a locked drawer and I could manage it myself. The nurses can be overrun |
|  |  | - If the nurses take over my medication and blood sugar testing |
|  |  | - Insulin on hand |
|  |  | - Machine to check sugar myself, medicines from nurse |
|  |  | - Manage my medications as I do at home with a Webster pack. |
|  |  | - Medicine in drawer |
|  |  | - Take my own insulins |
|  |  | - To have all my medications with me and take care of the medication myself |
|  |  | - Access to my own medication |
|  |  | - … having access to my own medications. |
|  |  | - Correct medication at correct time. |
|  | ***Assistance from hospital clinicians (eg endocrinologist, diabetic educator, nursing/midwifery staff)*** | |
|  |  | - It makes it easier with assistance from the endo team |
|  |  | - Access to resources like the diabetes educator |
|  |  | - Discussion with a diabetic educator |
|  |  | - I found the endo quickly checking in with me each day extremely helpful. |
|  |  | - Assistance of the nursing staff |
|  |  | - Help from nursing staff |
|  |  | - Better understanding of diabetes from my nurses |
|  |  | - The nurses like to make sure I was doing it right. |
|  | ***Checking blood sugar levels*** | |
|  |  | - [Having] my own gluco meter |
|  |  | - To [be able to] check my blood sugar in time |
|  |  | - [To be able to check my blood sugar levels] with my monitoring machine free Style lancets |
|  |  | - … bringing in my own bsl monitor to check. |
|  | ***Self-management of diabetes*** | |
|  |  | - Am so used to it |
|  |  | - I do exactly what I do at home |
|  |  | - Autonomy |
|  |  | - I suppose with some help |
|  |  | - if it is spiking the testing would be ok it depends of it if it is stable or not |
|  |  | - Keep a check on my diabetes as they have done |
|  |  | - More satisfying |
|  |  | - Myself |
|  |  | - If I am able I would like to be in charge of my own Diabetes but in saying that I’d like the nurses and doctors to be looking over to help out if I need help. |
|  | ***Appropriate food choices and timing*** | |
|  |  | - Better food |
|  |  | - Better food options, less questions from staff who don’t really understand diabetes. |
|  |  | - [Hospital staff] being more informed |
|  |  | - Having food that was more appealing to eat |
|  |  | - Having my medication & jelly beans & BSL tests with me |
|  |  | - I think at first I am just anxious, after a few days the BSL stabilizes itself. Meanwhile in hospital I tell my visitors not to bring in bananas or grapes and most of the time will not order desserts for the meal menu. |
|  |  | - Low GI food |
|  |  | - Menus including carb amounts |
|  |  | - More food options (more low GI and less starchy foods such as potatoes), more fresh fruit, salad and steam veggies options. Also Sugar free drinks |
|  |  | - Receiving meals and /or snacks at better times |
|  |  | - Wider variety of sugarless drinks and food, as most goods contained ingredients I am anaphylaxis to |
|  |  | - A choice needs to be offered when it comes to diabetic meals, myself and many others do not eat a diabetic diet at home we eat a regular diet and we would like to be offered that as an option rather than be forced to have the diabetic option. |
|  |  | - Fasting diabetic for a general anaesthetic forgetting to order breakfast so giving a juice jelly & chocolate mousse at 6am |
|  |  | - Food menus not having any carb details on them at all |
|  |  | - Meal time medication and taking reading won't coordinate properly. |
|  |  | - Missed snacks leading to hypos |
|  |  | - They [hospital staff] should listen more expressly when can’t eat |
| **Hospital diabetes care** | | |
|  | ***Satisfaction with diabetes care in hospital*** | |
|  |  | - All was managed well |
|  |  | - I was satisfied with the level of assistance |
|  |  | - Staff are very helpful |
|  |  | - Stay the same way |
|  |  | - They keep me reminding and update what is best and suitable for me |
|  |  | - I cannot think of anything as the nursing staff were very knowledgeable in the care of my diabetes. |
|  |  | - I could not of asked for better care they looked after me very well |
|  |  | - I was extremely pleased with my overall care whilst I was in hospital. The nurses were absolutely magnificent. In terms of diabetes knowledge though, I found most nurses didn’t have much of a clue about it. This didn’t impact me too much because I manage my own diabetes care and prefer it this way, and I took it as an opportunity to educate the staff on things like my insulin pump and continuous glucose monitor. It sometimes felt as though the staff were forced to check on my blood glucose levels and management rather than casually checking in and leaving me to my own devices. But overall I had a great experience at this hospital and the care I received. |
|  | ***Diabetes knowledge and expertise of hospital staff*** | |
|  |  | - A lot of the nursing staff didn't have a lot of knowledge about CGM and insulin pumps |
|  |  | - Definitely needs a look at more understanding when dealing with diabetics that need help not being made to give the control of their diabetes to the nurses that are not trained in living with diabetes |
|  |  | - Nurses generally just tick the boxes and rely on patient to explain everything. |
|  |  | - The level of care does often depend on the level of experience or expertise from the nurses taking care of you. Regular education to all members of the nursing team would be beneficial. Is there a yearly competency for staff caring for patients with a diabetes management plan? |
|  | ***Negative experiences of diabetes care in hospital*** | |
|  |  | - Being left to my own devices to manage my diabetes unless I ask for help. Not being put on a “diabetic menu”. Nurses being aware of the difference between the different types of diabetes. Nursing staff not so much as I felt they didn’t understand diabetes well enough. |
|  |  | - For one person to tell me what to do not 3 people |
|  |  | - FOR STAFF TO LISTEN TO ME |
|  |  | - Staff accepting that bsl is a moveable feast. Asking how my blood sugars are drives me nuts. Now or an hour ago. |
|  |  | - Because I was admitted by ambulance and not from home I was not prepared for admittance therefore I did not have my medication with me. As a result, after requesting Insulin I was not given any because they did not have Ryzodeg. I did explain that I could used Nova Rapid but they did not bring that either. I think they forgot. |
|  |  | - It took lots of arguments and a nurse screwing things up in order for me to be self-managed |
|  |  | - Doctors not always helpful |
|  |  | - Found night staff a bit ignorant & arrogant, glad I was sleeping most of the time. |
|  |  | - I discharged myself due to the ward nurse being offensive |
|  |  | - My last visit was a diabetic diagnosis and I was very sick (hyper). I was unaware of how to use insulin and was treated as type 1 until further testing came back. Little time and education given at start and things seemed rushed but lucky I was given a couple of extra days of education and until my blood sugar was more controlled |
|  |  | - Some nurses were diligent in checking my blood sugar levels, others didn't check it at all. None of the doctors wanted to discuss it at all. I had an argument every day with the nurses about what medication I was taking (or not taking). |
|  |  | - They were no follow up when I went home |
|  |  | - I am pretty competent in managing my diabetes and was not given the option to do so until after I had been there a few days |
|  |  | - Stop saying ‘poor control’ when hypos are the result of trying to determine insulin and carb needs when exercising. Makes me feel like l’m not doing a good job and l am not looking after myself properly or taking my diabetes management seriously, which l do every minute of the day! |
|  |  | - They changed my medication and put me on weekly injection. The injection made me very sick and lost a lot of weight. I was never overweight. The injection should not be given to those who do not have weight problem. More care should have been given. |
|  | ***Recommendations to improve diabetes care/management in hospital*** | |
|  |  | - CGM [continuous glucose monitor] |
|  |  | - Don’t take away my insulin, don’t put me on a restrictive diet, have an open understanding that we are not one size fits all. |
|  |  | - Education |
|  |  | - Extra help |
|  |  | - More education and information |
|  |  | - More encouragement |
|  |  | - More information about treatments given |
|  |  | - Nursing staff to trust that the patient is capable in managing their own diabetes and make sure supplies for insulin and hypos are accessible at all times. |
|  |  | - Support from staff to follow orders from doctors |
|  |  | - the nurses having completed notes |
|  |  | - Understanding by nurses |
|  |  | - To be listened to |
|  |  | - Be able to consult Diabetes educator if needed |
|  |  | - Would like to touch base with Endo while in patient |
|  |  | - Depends on the reasons I was in hospital. If medications or the diagnosis effected my diabetes I would want to know how to best manage all of the above. |
|  |  | - Depends on the situation; if I am unable to self-manage then will need assistance from nursing |
|  |  | - Each time I am hospitalized during the first days, my BSL is always between 11-16, so I prefer the nursing to assist me in controlling it, as it the lowers to around 7 |
|  |  | - Manage [diabetes] with staff |
|  |  | - Option of other food in recovery ie no white bread |
|  |  | - Happy to look after my own diabetes while an inpatient-if this is the case perhaps a subsidised CGM could be provided by hospital? Also would like an automatic visit from an endo anytime admitted |
|  |  | - I felt my nurses and midwives were really good this time with my diabetes and letting me being in control. So nice to see! |
